# Alcoholic and Non-Alcoholic Beer Modulate Plasma and Macrophage microRNAs Differently in a Pilot Intervention in Humans with Cardiovascular Risk

**DOI:** 10.3390/nu13010069

**Published:** 2020-12-28

**Authors:** Lidia Daimiel, Víctor Micó, Laura Díez-Ricote, Paloma Ruiz-Valderrey, Geoffrey Istas, Ana Rodríguez-Mateos, José María Ordovás

**Affiliations:** 1 Nutritional Genomics and Epigenomics Group, IMDEA Food, CEI UAM + CSIC, 28049 Madrid, Spain; victor.mico@imdea.org (V.M.); laura.diez@imdea.org (L.D.-R.); paloma.ruiz@imdea.org (P.R.-V.); jose.ordovas@tufts.edu (J.M.O.); 2 Department of Nutritional Sciences, School of Life Course Sciences, Faculty of Life Sciences and Medicine, King’s College London, London WC2R 2LS, UK; geoffrey.istas@kcl.ac.uk (G.I.); ana.rodriguez-mateos@kcl.ac.uk (A.R.-M.); 3 Nutrition and Genomics Laboratory, JM-USDA Human Nutrition Research Center on Aging at Tufts University, Boston, MA 02111, USA

**Keywords:** beer, biomarkers, cardiovascular disease, microRNAs, nutrition, inflammation

## Abstract

Beer is a popular beverage and some beneficial effects have been attributed to its moderate consumption. We carried out a pilot study to test if beer and non-alcoholic beer consumption modify the levels of a panel of 53 cardiometabolic microRNAs in plasma and macrophages. Seven non-smoker men aged 30–65 with high cardiovascular risk were recruited for a non-randomised cross-over intervention consisting of the ingestion of 500 mL/day of beer or non-alcoholic beer for 14 days with a 7-day washout period between interventions. Plasma and urine isoxanthohumol were measured to assess compliance with interventions. Monocytes were isolated and differentiated into macrophages, and plasma and macrophage microRNAs were analysed by quantitative real-time PCR. Anthropometric, biochemistry and dietary parameters were also measured. We found an increase in plasma miR-155-5p, miR-328-3p, and miR-92a-3p after beer and a decrease after non-alcoholic beer consumption. Plasma miR-320a-3p levels decreased with both beers. Circulating miR-320a-3p levels correlated with LDL-cholesterol. We found that miR-17-5p, miR-20a-5p, miR-145-5p, miR-26b-5p, and miR-223-3p macrophage levels increased after beer and decreased after non-alcoholic beer consumption. Functional analyses suggested that modulated microRNAs were involved in catabolism, nutrient sensing, Toll-like receptors signalling and inflammation. We concluded that beer and non-alcoholic beer intake modulated differentially plasma and macrophage microRNAs. Specifically, microRNAs related to inflammation increased after beer consumption and decreased after non-alcoholic beer consumption.

## 1. Introduction

Beer is a very popular beverage worldwide, and specifically in Europe. In 2016, 39 billion and 900 million litres of beer and non-alcoholic beer were produced in the European Union [1]. Beer is a fermented beverage with low alcohol content mainly composed of water, hops, yeast, and malted cereals (mainly barley, but it can also be produced with other cereals). Carbohydrates are the main nutrients of beer, but it is also rich in vitamins, minerals, and phenolic compounds [2,3,4]. Beer intake has been suggested to be beneficial for cardiovascular risk factors [5,6,7,8]. The mechanism behind this association could be the inhibition of the oxidation of low-density lipoproteins (LDLs), inducible nitric oxide synthase (iNOS), cyclooxygenase 1 and 2 (COX-1) and prostaglandin E [9,10]. However, most of these effects have been experimentally assayed in animal models, and with specific components of the beer, mainly polyphenols, and limited evidence in humans exists [7,8,9].

Beer is rich in polyphenols, approximately 70–80% of them coming from malt, and the remaining 30–20% coming from hops [11]. Within all beer polyphenols, 8-prenylnaringenin (8-PG) and isoxanthohumol (IX) and its derivatives are the most abundant [3]. 8-PG has been suggested to protect against type 2 diabetes mellitus (T2DM) elated metabolic dysfunctions in a mouse model [12], to show anticancer activity in U-118 MG cells [13], and to modulate the phosphorylation of PI3K, Akt, and P70S6K1 in myotube cells promoting a better recovery from muscle atrophy [14]. In addition, xanthohumol (a precursor of IX) reduces the growth of prostate tumours [15] as well as the accumulation of cholesterol in the aortic arch, increases HDL-cholesterol levels mediated by a CETP expression reduction [16], and ameliorates the atherosclerotic profile in mice models [17]. Xanthohumol also ameliorates metabolic syndrome, at least partly through mitochondrial uncoupling and stress response induction [18]. These results suggest that the main beer polyphenols affect metabolic and signalling pathways related to cardiovascular disease (CVD). However, the effect of the entire beverage has not been studied in-depth, and the molecular mechanisms mediating the effects of beer intake are poorly understood.

Studies conducted in pigs have shown that both beer and non-alcoholic beer improve the effect of vasodilatory drugs and prevent the endothelial dysfunction associated with a Western diet [10]. Moreover, beer and non-alcoholic beer-fed animals showed a better recovery and smaller scar size after the induction of myocardial infarction, with slightly better results observed in pigs fed with beer compared with those fed with non-alcoholic beer [19]. The effect of beer and non-alcoholic beer on endothelial function in the pig model could be explained by a decrease in oxidative damage, an increase in Akt activation, a decrease in apoptosis and lipid peroxidation in the ischaemic area and an increase in collagen deposition during scar formation [10,19]. These studies support a potential cardioprotective effect of beer. Due to the low alcohol content, beer can be ingested in moderate amounts and, given its nutritional and caloric profile, beer can be included in a healthy dietary pattern. However, further analyses are needed to fully elucidate the effect of beer intake, both alcoholic and non-alcoholic beer, in humans. In this regard, the study of how beer intake modulates microRNAs with a well-known role in CVD will increase the knowledge of the impact of beer on human health.

MicroRNAs are short (22–24 nucleotides) non-coding sequences that modulate many cellular processes by regulating the expression of their target genes [20]. They have been shown to influence cholesterol metabolism [21], T2DM [22], heart stroke [23], insulin sensitivity [22], or endothelial function and inflammation [21]. Most of these processes are related to CVD [24]. In addition, microRNAs have been found in plasma and other biofluids [25]. The discovery of circulating miRNAs opened a new way as they can be studied as biomarkers of diseases or nutritional status [26].

In this study, we aimed to investigate the modulation of circulating microRNAs and microRNAs isolated from macrophages after intake of beer and non-alcoholic beer. The aim was to analyse whether this modulation was associated with CVD risk and discern the molecular processes affected by such modulation in subjects with high CVD risk.

## 2. Materials and Methods

### 2.1. Population and Study Design

The miRoBeer study was a controlled, cross-over clinical trial with beer and non-alcoholic beer. Seven volunteers, men aged 30–65 years, were recruited at Instituto Madrileño de Estudios Avanzados, IMDEA Food Institute with the following inclusion criteria: non-smokers, with two or more cardiovascular risk factors such as familial history of early coronary disease (documented myocardial infarction or sudden death before 55 years of age in first-degree male relatives or before 65 years of age in first-degree female relatives), hypertension (systolic blood pressure ≥ 130 mm Hg or diastolic blood pressure ≥ 85 mm Hg or taking antihypertensive medication), BMI ≥ 25 kg/m^2^, HDL-cholesterol ≤ 40 mg/dL, LDL-cholesterol ≥ 160 mg/dL, triglycerides (TG) > 150 mg/dL. Exclusion criteria were: previous CVD, HIV infection, alcoholism or history of abuse of other toxic substances, BMI ≥ 40 kg/m^2^, hypersensitivity or allergy to any beer compounds, intestinal diseases, illiteracy, or any other disease or condition that could worsen compliance [27]. The study was conducted at IMDEA Food Institute (Madrid, Spain) from October 2015 to December 2015 (Figure S1a).

The intervention consisted of two beer/non-alcoholic beer intake periods of 14 days, preceded by two washout periods of 7 days (Figure S1b). Participants were not allowed to consume any other fermented beverage except for the beer we provided during the intervention periods, while no fermented beverages were allowed during washout periods. A total of 500 mL of lager beer was ingested daily. Beer was produced in Spain and was provided by Centro de Información Cerveza y Salud through the Asociación de Cerveceros de España (Fundación Cerveza y Salud). Beer ingestion was not limited to a specific time of the day or a specific number of doses per day. Blood and urine samples were collected after each washout and intervention period according to the experimental design shown in Figure S1.

During the study, any change in dietary habits was monitored through a 3-day dietary record (two weekdays and one weekend day) taken at each visit. Participants were appropriately trained to correctly record the 3-day dietary record by trained dietitians. Any excessive alcohol intake was detected and monitored with a CAGE (cut-annoyed-guilty-eye) questionnaire. Trained dietitians recorded the CAGE questionnaire at each visit.

The Ethics Committee of IMDEA Food approved the intervention protocol (ref. IMD PI-0016), and all volunteers provided written informed consent. All procedures were performed following the 1964 Helsinki Declaration and its late amendments.

### 2.2. Dosage Information

The volunteers ingested 500 mL of beer or non-alcoholic beer. This dose of beer ingested in one or several doses per day with meals did not result in adverse events. The intake of non-alcoholic beer is not associated with adverse events due to its negligible alcoholic content. It has been stated that alcohol consumption below 30 g/day, corresponding to up to two alcohol units per day, reduces cardiovascular risk [6]. Two units of alcohol per day for beer correspond to 660 mL/day (2 × 330 mL/unit). The used dosage of beer, 500 mL/day, contains approximately 0.054 g of alcohol per mL. Thus, the ingestion of 500 mL of beer in one day supposes 27 g, below the safe threshold. Participants could ingest the beer in one or more doses per day and at any hour of the day, and they were advised to drink the beer with meals. During the intervention, no other fermented drinks were allowed and the intake of any other alcoholic beverage (distilled drinks) during the trial was discouraged.

### 2.3. Anthropometric, Body Composition and Dietary Measurements

Anthropometric measures and body composition were recorded at each visit. Height was measured in a wall-mounted stadiometer, and weight was measured with light clothes with an Omron BF 511 calibrated scale (OMRON Healthcare Europe, Hoofddorp, Netherlands). BMI was calculated as weight in kilograms divided by the square of height in metres. Fat mass (%), muscle mass (%), resting metabolic rate (RMR) (kcal) and visceral fat index were measured by bioimpedance with the Omron BF 511 calibrated scale (OMRON Healthcare Europe, Hoofddorp, Netherlands).

Energy and nutrient intake was calculated as the average frequency of intake of the three recorded days multiplied by the nutrient composition of the specified portion size for each food item with DIAL software, based on available information on food composition [28].

### 2.4. Collection of Blood and Urine Samples

Blood was collected after 12-h fasting by a venous puncture in 10 mL Vacutainer tubes with K_2_EDTA anticoagulant. Blood samples were centrifuged at 1500× *g* for 15 min and 4 mL of plasma were finally recovered. A spot urine sample was collected in the morning. All samples were stored at −80 °C until processing. Biochemistry analyses, including lipid profile and C-reactive protein (PCR), were carried out by CQS Laboratories (Madrid, Spain) using standard procedures [27].

### 2.5. Peripheral Blood Mononuclear Cells (PBMCs) Isolation and Macrophage Selection

Blood was collected as described above and whole blood was mixed with 10 mL of RPMI medium (Cultek, Madrid, Spain) supplemented with L-glutamine (Cultek, Madrid, Spain) and 10% foetal bovine serum (FBS) (Gibco, Invitrogen, Carlsbad, CA, USA) and then mixed with an equal volume of LYMPHOPREP^TM^ (STEMCELL Technologies, Grenoble, France). The mixture was centrifuged at 900 g for 40 min, and the cellular layer containing PBMCs was collected and washed three times with phosphate-buffered saline (PBS) (Gibco, Invitrogen, Carlsbad, CA, USA). The purified PBMCs were cultured in RPMI complete medium in a non-treated plate and incubated at 37 °C 5% CO_2_ conditions for 4 h. After that, adherent cells (macrophages) were picked and cryopreserved in RPMI medium with 10% dimethyl sulfoxide (DMSO) (Scharlau, Barcelona, Spain) and 11.25% FBS. Cryopreserved cells were stored in liquid nitrogen until use [27].

### 2.6. 8-PG and IX Quantification

Beer, plasma, and urine 8-PG and IX were analysed from 500 µL of plasma and urine and 50 µL of beer, using a validated method with some modifications [28]. Plasma and urine were treated with a mix of β-glucuronidase and arylsulfatase with a final specific activity of 81,700 IU of β-glucuronidase and 700 IU of arylsulfatase to liberate any 8-PG and IX conjugated. Samples were then centrifuged at 15,000× *g* for 15 min at 4 °C and 353 µL of the supernatant was diluted (1:1) with phosphoric acid 4% and spiked with a standard mix (50 nM) as an internal standard. 600 µL were loaded on a 96-well µ-SPE HLB plate, washed with 200 µL of water and 200 µL of 0.2% acetic acid and finally eluted with 60 µL of methanol. Extracted and concentrated plasma samples were purified with a solid-phase extraction using Oasis^®^ HLB µElution plate. This process allows five-times concentration of the initial sample to improve polyphenols detection. A calibration curve of 8-PG and IX was performed using internal standards. Analyses were carried out using a Thermo Scientific™ Exactive™ Plus Orbitrap (Thermo Scientific, Waltham, MA, USA). The results were analysed using Xcalibur software [27].

### 2.7. MicroRNA-Enriched RNA Isolation

MicroRNA-enriched total RNA from plasma and macrophages was isolated with miRCURY™ RNA Isolation Kit-Biofluids (Exiqon, Madrid, Spain) and miRCURY™ RNA Isolation Kit-Cell & Plant (Exiqon, Madrid, Spain), respectively. The extracted RNA was quantified using NanoDrop 2000 (Isogen LifeSciences, Utrecht, Netherlands).

### 2.8. MicroRNAs Expression Analysis

We measured plasma and macrophage expression levels of a panel of 53 microRNAs as previously described [27,29]. We used a customised 56-probes OpenArray plate (Thermo Fisher Scientific, Waltham, MA, USA). We included three spike-in controls in our customised OpenArray plate. Therefore, a panel of 53 microRNAs related to CVD was selected. They were selected using prediction algorithms such as miRWalk [30] and miRbase [31] and a literature search (Table S1). Briefly, 5 µL of RNA from plasma and 50 ng of RNA from macrophages were retro-transcribed with the TaqMan^®^ MicroRNA Reverse Transcription Kit (Thermo Fisher Scientific, Waltham, MA, USA). Before retro transcription, 5fmol of single-strand cel-miR 54 was added to control for retro transcription variability. Resulting cDNA was pre-amplified with TaqMan^®^ PreAmp Master Mix (Thermo Fisher Scientific, Waltham, MA, USA). Preamplification product was diluted 1:20 in plasma samples and 1:40 in macrophage samples. A total of 3 µL of diluted preamplification product was combined with TaqMan OpenArray PCR Master Mix (Thermo Fisher Scientific, Waltham, MA, USA) and loaded onto a TaqMan custom OpenArray plate using an OpenArray^®^ AccuFill™ System (Thermo Fisher Scientific, Waltham, MA, USA). The qPCR was run in the QuantStudio™ 12K Flex Real-Time PCR System with OpenArray^®^ Block (Thermo Fisher Scientific, Waltham, MA, USA).

The fold change (FC) in circulating and macrophage microRNAs levels was measured as relative quantification using the 2^−ΔΔCt^ method, comparing each intervention point with its corresponding basal point, and was Log_2_ transformed. Normalisation and relative quantification analyses were described previously [27,29]. Briefly, the NormFinder algorithm [32] was used to select the best endogenous reference microRNAs in each case (miR-24-3p for plasma and miR-146a-5p and miR-320a-3p for macrophages). The quadratic average of the endogenous controls plus the exogenous cel-miR-54 control was used for normalisation. The efficiency of all probes was assessed with a calibration curve using a control RNA from plasma. All microRNAs with an efficiency below 80% were discarded. Two non-template controls (NTCs) were included in each plate, and all microRNAs with CTs over the NTC were also discarded. RT-qPCR was made in duplicate, and all microRNAs with >10% of inconsistent technical replicates were discarded.

### 2.9. RNA Isolation and Gene Expression Analysis

RNA from PBMCs was isolated with miRCURY^TM^ RNA Isolation Kit-Cell & Plant (Exiqon, Aarhus, Denmark) following manufacturer’s instructions. RNA was quantified with a NanoDrop 2000 and integrity was assessed in a 2% agarose gel. A total of 500 µL of RNA were retro-transcribed with PrimeScript Reverse Transcription Kit (Takara, Saint-Germain-en-Laye, France) according to manufacturer’s protocol and resulting cDNA was used as a template for amplifying *MDM2* and *DENND5B* genes by RT-qPCR with FastStart Universal SYBR Green Master. Primers used for *MDM2* amplification were 5′-CCCAAGACAAAGAAGAGAGTGTGG-3′ and 5′-CTGGGCAGGGCTTATTCCTTTTCT-3′ forward and reverse primers, respectively. To amplify *DENND5B*, we used 5′-CTCCAGCGATACAACTCCTATGA-3′ and 5′-GTGGATATAGCTTTCAAGTGGCA-3′ forward and reverse primes, respectively. Relative gene expression was calculated with the 2^−ΔΔCt^ method using the quadratic average of RN18S and RPLP0 CTs as references.

### 2.10. In Silico Functional Analyses

MicroRNAs significantly modified in plasma and macrophages were selected for further functional analyses. We performed an enrichment analysis as previously described [27,29], using DIANA miRPath v0.3 [33] to identify KEGG (Kyoto Encyclopedia of Genes and Genomes) pathways and GO (Gene Ontology) biological processes associated with modified microRNAs and miRWalk to search for experimentally validated and predicted targets of these microRNAs using [29]. We compared eight algorithms (miRanda, TargetScan, miRDB, PITA, MicroT4, miRMap, miRNAMap and miRWalk) using stringent criteria in all of them. Targets predicted by at least seven algorithms were selected for further analysis. Both experimental and predicted targets were analysed for functional GO annotation terms and KEGG pathways using Babelomics 5 and Software String and applying a false discovery rate (FDR) [34,35].

### 2.11. Statistical Analyses

FCs for all microRNAs in each timepoint were log2 transformed for normalisation. Changes among conditions were tested with a repeated measurement ANOVA test including a Mauchly’s sphericity test. We additionally carried out a Kolmogorov-Smirnov test for normal distribution. For the modified microRNAs in both plasma and macrophages, FC after beer intake was compared with its corresponding washout point with paired *t*-test or the corresponding non-parametric test. The FCs of the two washout timepoints were additionally compared to discard the persistence of an effect of the previous treatment. A two-tailed *p*-value of 0.05 was considered significant. IX and 8-PG levels are shown as the mean ± standard deviation (SD). Average IX and 8-PG levels were compared among intervention phases with ANOVA. Statistical analyses were carried out with SPSS v24.

Statistical significance for enriched GO biological processes and KEGG pathways was analysed with Babelomics 5, Diana miRPath v0.3, and Software String algorithms applying an FDR adjustment. Overrepresented KEGG pathways are shown in heat maps with the colour representing the log_10_ of the *p*-value or in bar graphs. Venn diagrams were built with the Bioinformatics and Evolutionary Genomics application using default settings (http://bioinformatics.psb.ugent.be/webtools/Venn/) [27,29].

Correlation between the FC of plasma and macrophage microRNAs was analysed by Tau Kendal test. Correlation between microRNAs levels and anthropometric and biochemistry parameters and the correlation between the FC in circulating microRNA levels and the FC in plasma IX and 8-PG levels were analysed by Pearson correlation test. Correlation analyses and scatterplots were created in R i386 3.5.2 using RStudio. Heat map correlation plots were created using the Corrplot package in R i386 3.5.2 using RStudio.

## 3. Results

### 3.1. Characteristics of the Population

Table 1 shows the characteristics of the population in each experimental timepoint (Table 1). Characteristics of the population did not change along the intervention, except for a decreased mono-unsaturated fatty acid (MUFA) consumption in each intervention period (*p* = 0.02) (Table 1).

### 3.2. Beer Polyphenol Measurements

IX has been described as a sensitive and specific urine marker of beer consumption [36]. We measured plasma and urinary levels of IX and 8-PG to test adherence to the intervention. We first measured these two polyphenols in the samples of beers provided to the participants (Figure S2). We observed that IX concentration was higher in beer, while 8-PG concentration was higher in non-alcoholic beer. These results suggest that the nutritional composition of beer can change during the dealcoholising procedure.

**Figure 1:**
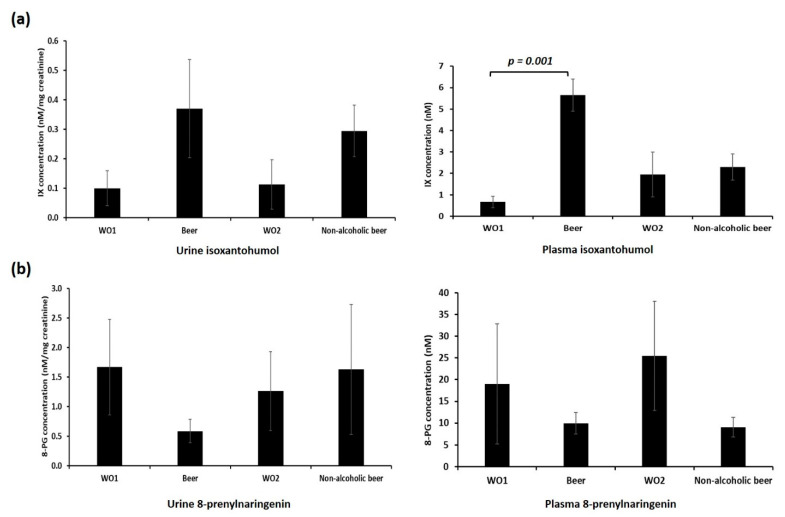
Plasma and urine (**a**) isoxanthohumol (IX) and (**b**) 8-prenylnaringenin (8-PG) levels. Plasma and urine IX and 8-PG levels were measured by mass spectrometry. *p*-value refers to the intra-subject comparison of the paired Student’s *t*-test. WO1: washout 1, Beer: alcoholic beer intervention; WO2: washout 2, non-alcoholic beer: non-alcoholic beer intervention. Data represent mean ± SD.

We then measured 8-PG and IX in plasma and urine samples of each volunteer at each time point (Figure 1). As expected, urine IX concentration was higher after each intervention period compared with both washout periods (10.9-fold and 3.71-fold increase after beer and non-alcoholic beer intake, respectively) (Figure 1a, left panel). We observed a similar increase in plasma IX concentration after beer intake (an 8.5-fold increase compared with basal time), but not after non-alcoholic beer intake (1.18-fold increase compared with previous washout time) (Figure 1a, right panel). Therefore, we considered that participants had adhered effectively to the intervention and that the washout periods had been effective.

We did not observe an increase in urine or plasma 8-PG levels after beer or non-alcoholic beer consumption. We found a non-significant decrease in urine 8-PG levels after beer consumption and a non-significant decrease in plasma after both beer and non-alcoholic beer consumption (Figure 1b). These results suggest that 8-PG is not a useful biomarker of beer intake, and further studies are needed to elucidate the mechanism behind the observed decrease of 8-PG after beer intake.

### 3.3. Effect of Beer and Non-alcoholic Beer Consumption on Circulating and Macrophage Levels of microRNAs

**Figure 2:**
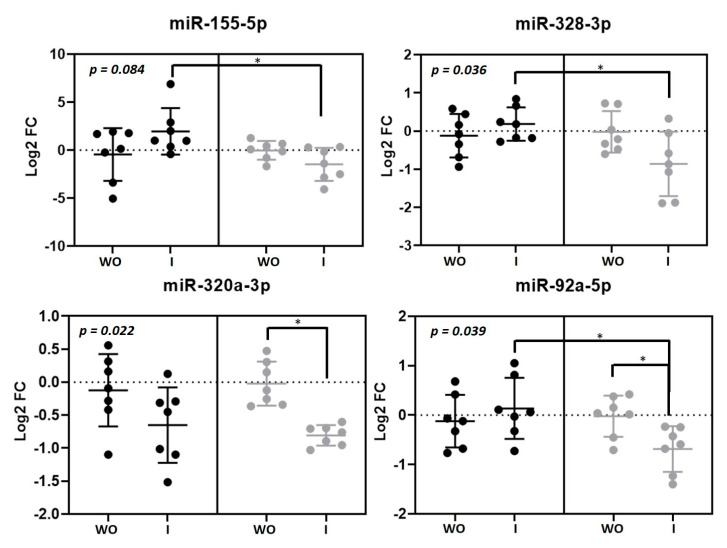
Circulating microRNAs modulated by beer and non-alcoholic beer. Scatterplots showing Log_2_ transformed relative fold change (FC) microRNAs in each intervention phase (*n* = 7). Black dots (●) correspond to beer intervention and grey dots (●) correspond to non-alcoholic beer intervention. Plasma microRNA levels were calculated with the 2^−ΔΔCt^ method comparing with each basal timepoint. Mean ± SD of each timepoint is shown. *p*-value was calculated with repeated measurements ANOVA. * *p* < 0.05 for the paired *t*-test comparison between timepoints. WO: washout; I: intervention.

Plasma expression levels of four microRNAs were significantly changed by the intervention (Figure 2). MiR-155-5p (*p* = 0.036), miR-328-3p (*p* = 0.036) and miR-92a-3p (*p* = 0.039) were upregulated after beer intake and downregulated after non-alcoholic beer intake. We found that FC after beer intake was significant in comparison with FC after non-alcoholic beer intake. MiR-320a-3p was downregulated after both beer and non-alcoholic beer intake (*p* = 0.022). We found an interesting positive correlation between miR-320a-3p and LDL-cholesterol plasma levels (*R* = 0.45, *p* = 0.027) (Figure S3).

**Figure 3:**
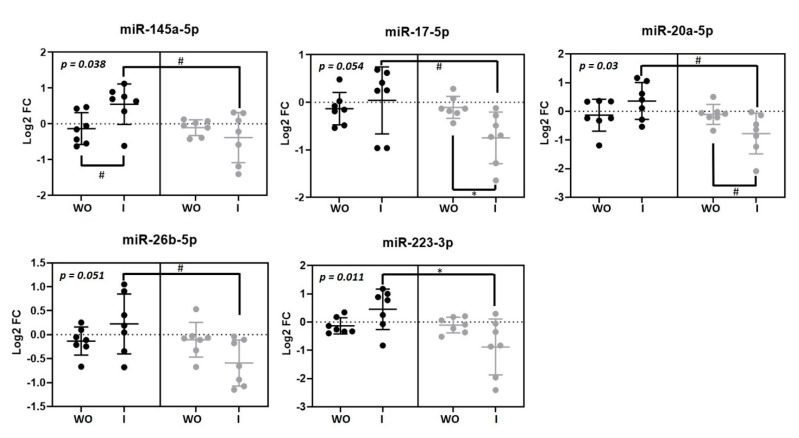
Macrophage microRNAs modulated by beer and non-alcoholic beer. Scatterplots showing Log_2_ transformed relative fold change (FC) microRNAs in each intervention phase (*n* = 7). Black dots (●) correspond to beer intervention and grey dots (●) correspond to non-alcoholic beer intervention. Macrophage microRNA levels were calculated with the 2^−ΔΔCt^ method comparing with each basal timepoint. Mean ± SD of each timepoint is shown. *p*-value was calculated with repeated measurements ANOVA. WO: washout; I: intervention. * *p* < 0.05; # *p* < 0.1.

Five microRNAs were modulated by beer intake in macrophages miR-145-5p (*p* = 0.038), miR-17-5p (*p* = 0.05), miR-20-5pa (*p* = 0.03), miR-26b-5p (*p* = 0.05) and miR-223-3p (*p* = 0.011) (Figure 3). Interestingly, all of them showed the same response as they were upregulated after beer consumption and downregulated after non-alcoholic beer consumption.

### 3.4. Correlation between Circulating microRNA Levels and Macrophage microRNA Levels

**Figure 4:**
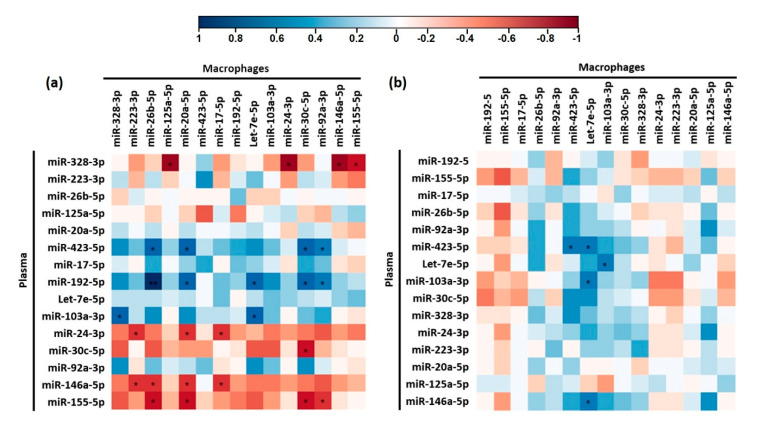
Heat map correlation plots of plasma and macrophage microRNAs modulated beer (**a**) and non-alcoholic beer (**b**). Correlation was calculated by Tau Kendall test. * *p* < 0.05. Blue cells represent positive correlations while red cells represent negative correlations. The intensity of the colour represents the strength of the correlation according to the indicated colour key.

We aimed to identify correlations between changes in plasma and macrophage microRNAs to test if plasma levels of some microRNAs were potential biomarkers of their levels in macrophages (Figure 4). We found that FC in plasma and macrophage miR-30c-5p levels after beer intake was strongly negatively correlated (*R* = −0.81, *p* = 0.011) (Figure 4a). We observed two different correlation clusters in beer intervention. One of them included plasma microRNAs whose FC positively correlated with the FC of different macrophage microRNAs: miR-423-5p, miR-192-5p, and miR-103a-3p. Another cluster included plasma microRNAs whose FC correlated negatively with the FC of other macrophage microRNAs: miR-24-3p, miR-146a-5p, and miR-155-5p (Figure 4a). Only plasma FC of miR-423-5p correlated with its macrophage FC after intake of non-alcoholic beer (*R* = 0.714, *p* = 0.03) (Figure 4b).

### 3.5. Functional Analyses

**Figure 5:**
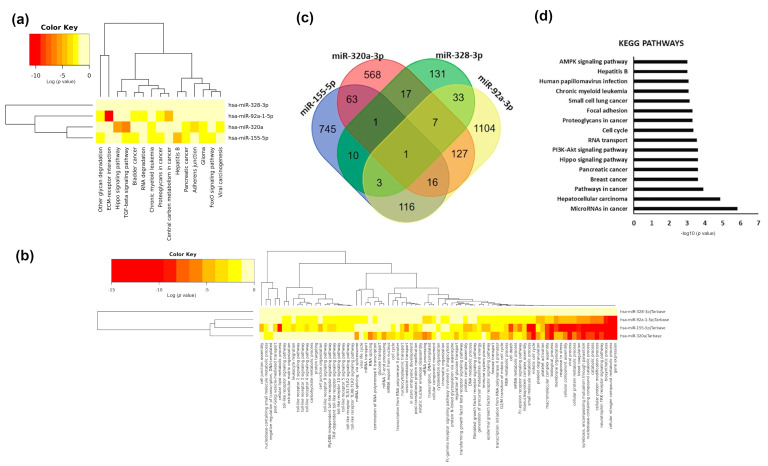
Functional analyses of modified plasma microRNAs. (**a**) Cluster heat map of KEGG pathways significantly enriched among modulated plasma microRNAs. (**b**) Cluster heat map of GO biological processes significantly enriched among modulated plasma microRNAs. The colour bar represents –log_10_ of the enrichment *p*-value after FDR correction. (**c**) Venn diagram showing common targets of the modulated microRNAs. (**d**) Bar chart of the most represented KEGG pathways (FDR adjusted *p*-value < 0.001) of the predicted and validated targets of modified plasma microRNAs. X-axis represent –log_10_ of the enrichment *p*-value after FDR correction.

We further carried out a bioinformatic analysis to identify experimental and validated targets of the modulated microRNAs and performed functional enrichment analyses to identify the main biological processes and molecular pathways where the modified microRNAs play a role. We found that plasma modulated microRNAs were mainly involved in cancer, although miR-155-5p and miR-320a-3p were also related to TGFβ signalling (Figure 5a). miR-320a-3p and miR-155-5p share several pathways, suggesting that the concomitant decrease in plasma levels of both microRNAs can specifically impact on those pathways. We also observed a cluster of biological processes associated with miR-155-5p, miR-320a-3p, and miR-92a-3p. They are mainly involved in gene expression and catabolism.

Interestingly, miR-92a-3p and miR-155-5p are both associated with Toll-like receptors signalling and lipid metabolic processes (Figure 5b). We searched for validated and predicted targets of two out the four plasma microRNAs modulated by beer and found 401 genes commonly targeted (Figure 5c). Functional analyses of those genes showed that they were related to different types of cancer and play a role in related processes (cell cycle progression). They were also related to nutrient-sensing pathways such as PI3K-AKT or AMPK (AMP-activated kinase) pathways (Figure 5d). Only one gene, *SOX11* (SRY-box 11), was the target of the four modulated plasma microRNAs (Table S2). Only one gene, *TMOD3* (tropomodulin 3), was the target of miR-155-5p, miR-320a-3p and miR-328-3p (Supplementary Table S2). Finally, three genes: SLC7A1 (solute carrier family 7 member 1), PTPRJ (protein tyrosine phosphatase receptor type 1) and SCD (stearoyl-CoA desaturase) were targets of miR-155-5p, miR-328-3p and miR92a-3p (Table S2).

**Figure 6:**
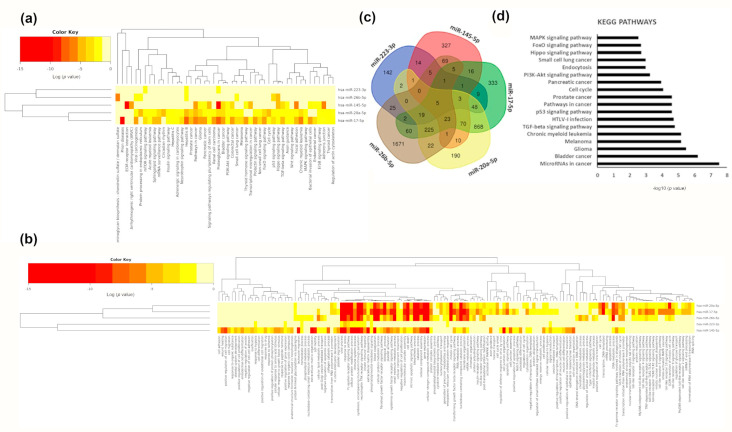
Functional analyses of modified macrophage microRNAs. (**a**) Cluster heat map of KEGG pathways significantly enriched among modulated macrophage microRNAs. (**b**) Cluster heat map of GO biological processes significantly enriched among modulated macrophage microRNAs. The colour bar represents –log_10_ of the enrichment *p*-value after FDR correction. (**c**) Venn diagram showing common targets of the modulated microRNAs. (**d**) Bar chart of the most represented KEGG pathways (FDR adjusted *p*-value < 0.01) of the predicted and validated targets of modified macrophage microRNAs. X-axis represents –log_10_ of the enrichment *p*-value after FDR correction.

Among pathways regulated by macrophage modulated microRNAs, we found different types of cancers. However, we also found enrichment in signalling pathways such as MAPK, Wnt, TGF-beta, Hippo, p53, FoxO, prolactin, PI3K/AKT, thyroid hormone, neurotrophin, insulin and mTOR (Figure 6a). As expected, miR-17-5p and miR-20a-5p, members of the same cluster, shared most pathways. Additionally, miR-145-5p was associated with ECM (extracellular membrane) receptor interaction, focal adhesion, and adherents’ junctions as well as arrhythmogenic right ventricular cardiomyopathy (Figure 6a). Interestingly, miR-20a-5p was also associated with circadian rhythm (Figure 6a). When we analysed GO biological processes, we found a cluster of processes related to the immune system and catabolic processes shared by all microRNAs except for miR-223-3p (Figure 6b). Finally, miR-17-5p, miR-20a-5p, and miR-26b-5p were all associated with Toll-like receptors signalling (Figure 6b). A total of 1729 genes were common targets of at least three out of five of the microRNAs significantly modulated by beer intake in macrophages (Figure 6c). All five modulated microRNAs targeted five genes: *MDM2* (*MDM2* Proto-Oncogene), *DENND5B* (DENN Domain Containing 5B), *ANKRD52* (Ankyrin Repeat Domain 52), *PURB* (Purine Rich Element Binding Protein B) and *ST8SIA3* (ST8 Alpha-N-Acetyl-Neuraminide Alpha-2,8-Sialyltransferase 3) (Table S3). Targeted genes were related to cancer and nutrient-sensing pathways like the PI3K-AKT and AMPK signalling pathways (Figure 6d). PKFB2 (6-phosphofructo-2-kinase/fructose-2,6-biphosphatase 2), with a role in nutrient sensing, was the target of four modulated macrophage microRNAs (miR-17-5p, miR-20a-5p, miR-223-3p and miR-26b-5p). We also observed that genes involved in lipid metabolism like *ABCA1* (ATP Binding Cassette Subfamily A Member 1) or *ACSL4* (Acyl-CoA Synthetase Long-Chain Family Member 4) were targets of miR-145-5p, miR-17-5p, miR-20a-5p and miR-26b-5p (Table S3). We analysed the expression of *DENND5B* and *MDM2* in macrophages in all conditions. Interestingly, although no significant changes were found, mainly due to the small sample size and the big variability, we found that both genes were more upregulated after non-alcoholic beer intake than after beer intake (Figure S4). These results are concordant with the downregulation of microRNAs targeting these genes after non-alcoholic beer intake.

### 3.6. Correlation between Changes in Circulating microRNAs and Plasma IX Levels after Beer and Non-alcoholic Beer Intake

We then aimed to identify correlations between the FC of IX and 8-PG in plasma and the FC of identified circulating microRNAs (Figure S5). We found a negative correlation between FC in circulating levels of miR-155-5p (*R* = −0.84, *p* = 0.019) and miR-92a-5p (*R* = −0.82; *p* = 0.023) and FC in IX levels after the intervention with non-alcoholic beer. We did not find any other significant correlation between changes in circulating microRNAs and IX or 8-PG levels after beer or non-alcoholic beer intake.

## 4. Discussion

Some evidence about the impact of beer consumption on cardiovascular health has been reported [10,19], although molecular mechanisms underlying such an impact are not known. We hypothesised that beer and non-alcoholic beer consumption could modify the expression of microRNAs related to CVD. To test this hypothesis, we carried out a pilot intervention study to identify circulating and macrophage microRNAs modulated by beer and non-alcoholic beer intake. For this purpose, we selected a panel of 53 microRNAs related to CVD, and we measured their expression levels in plasma and blood-derived macrophages using a custom 56-probes OpenArray Plate (Thermo Fisher Scientific, Waltham, MA, USA). Our main finding related to the differential effect of both types of beer on the expression of modulated microRNAs. In general, beer increased while non-alcoholic beer decreased the expression of the modulated microRNAs. To our knowledge, this is the first study showing an effect of beer intake on the expression of microRNAs.

Circulating miR-155-5p, miR-328-3p and miR-92a-3p levels were increased after beer consumption and decreased after non-alcoholic beer consumption. Circulating levels of miR-328 were higher in patients of acute myocardial infarction compared to healthy controls [23] and were associated with atrial fibrillation and dilatation [37]. This miRNA modulated atrial electrical remodelling through regulation of L-type Ca (2+) channel density [38]. Plasma levels of miR-328 were also decreased by long-term treatment with ω-3 and ω-6 polyunsaturated fatty acids [39]. miR-155 has been recognised as a pro-inflammatory microRNA [40] and circulating levels of this microRNA were altered in patients with coronary artery disease [41,42]. Inhibition of miR-92a was associated with better remodelling after myocardial infarction [43]. miR-92a is a member of the miR-17-92 cluster. The increase in the expression of this cluster, also known as oncomiR-1, was associated with different types of cancer, and it was suggested to be a key regulator of tumour cell metabolism [44]. Specifically, the overexpression of this cluster enhanced glycolysis and oxidative phosphorylation in tumour cells, with different members of the cluster having different effects [44]. In this regard, the lack of miR-92a was found to increase glycolytic and oxidative metabolism [44], likely through the regulation of the PTEN/AKT signalling pathway [45]. The downregulation of this cluster was suggested to be a biomarker of healthy aging [46] and was found in Rhesus monkeys under a caloric restriction, a well-known intervention to promote longevity [47]. miR-92a was also suggested to be involved in endothelial cell macrophage communication. In this regard, it was reported that endothelial cells excrete miR-92a within vesicles that are captured by macrophages, promoting an atherogenic phenotype of macrophages [48]. On the contrary, downregulation of miR-92a decreased the expression of inflammatory cytokines in macrophages [48]. Moreover, inhibition of miR-92a improved endothelial cell regeneration and reduced restenosis in vascular injury via regulation of KFL4 and MMK4 gene expression [49]. Therefore, the downregulation we observed after non-alcoholic beer intake could suggest that this microRNA was mediating a potential cardioprotective effect of non-alcoholic beer. Circulating levels of miR-320 were decreased by both beer and non-alcoholic beer, with a more significant effect of non-alcoholic beer. Interestingly, circulating miR-320 levels decreased in diabetic subjects when compared with age- and sex-matched controls [50], but overexpression of miR-320 promoted cardiomyocyte apoptosis and the loss of mitochondrial membrane potential in mice [51,52] and the reduction of neointimal formation through the reduction of vascular smooth muscle cell proliferation and migration [53]. It was proved that miR-320 is enriched in exosomes released by cardiomyocytes of diabetic rats and that miR-320 is transferred from exosomes to cardiac endothelial cells where they inhibited proliferation, migration, and tube-like formation [54]. We found that plasma miR-320 levels positively correlated with LDL levels, suggesting that lower circulating miR-320 is associated with a better plasma lipid profile, supporting a potential role of circulating miR-320 as a biomarker of CVD. As far as we know, this is the first study reporting such correlation in humans, although Chen et al. showed that higher miR-320a expression resulted in higher fasting triglycerides, total cholesterol and LDL-cholesterol levels and lower fasting HDL-cholesterol levels in a mouse model of atherosclerosis [55]. In general, the decrease in plasma levels of these microRNAs after non-alcoholic beer intake suggests a better cardiovascular, endothelial function and aging profile. According to our in silico analyses, this suggested a better cardiovascular profile after non-alcoholic beer intake, which can have an impact on catabolism, nutrient sensing, and Toll-like receptors signalling.

Moreover, only one gene was the target of all these microRNAs: *SOX11* (*SRY-Box 11*). *SOX11* is a transcription factor involved in the regulation of embryonic development and in determining cell fate and has been related to tumorigenesis [56]. *TMOD3* was targeted by miR-155-5p, miR-320a-3p and miR-328-3p. Both are related to cardiac development, cardiomyopathy, and cardiac conduction through the ERK pathway [57,58,59,60,61]. Our study suggested that beer and non-alcoholic beer consumption modulated the expression of microRNAs involved in cardiac remodelling and that such modulation could be mediated by their targeting of these genes. Nevertheless, further studies are needed to elucidate the impact of the modulation of these circulating microRNAs on signalling pathways in different target tissues.

Similarly, we found that miR-145-5p, miR-17-5p, miR-20a-5p, miR-26b-5p, and miR-223-3p were upregulated in macrophages after beer intake and downregulated after non-alcoholic beer intake. Both miR-17 and miR-20a belong to the same cluster as miR-92a. These results suggested that this cluster could be a key mediator of the effect of beer intake on cardiovascular health. Moreover, it was shown that this cluster plays a crucial role in macrophage differentiation [62]. Liang et al. described the *ABCA1* targeting by miR-20a and the consequent impact on cholesterol efflux in macrophage cell lines [63]. Our functional analyses also showed that *ABCA1* was a predicted target of miR-17-5p, miR-20a-5p, miR-26b-5p, and miR-145-5p. miR-18a and miR-19, other microRNAs of the same cluster, were upregulated in peripheral mononuclear cells 2 h after a fat load [64] and in obese individuals after eight weeks following an intervention based on an isocaloric diet enriched in ω-3 and ω-6 polyunsaturated fatty acids [39]. These two microRNAs were not in our panel. Thus, we cannot discard their modification by beer intake. Lack of miR-17 and miR-20a was shown to reduce glycolytic and oxidative metabolism in tumour cells [44] and increase AMPK and decrease mTOR signalling pathways [44]. Our functional enrichment analyses also showed that these nutrient-sensing pathways were significantly targeted by these microRNAs, suggesting that beer and non-alcoholic beer could differentially modulate these pathways through miR-17 and miR-20a. Circulating miR-26b levels were higher in hypertensive patients with left ventricular hypertrophy compared with hypertensive patients without left ventricular hypertrophy and healthy controls [65]. On the other hand, circulating miR-26b levels were higher in ST-segment elevation myocardial infarction patients who suffered a major cardiovascular event after one year of follow-up [66]. Therefore, circulating levels of miR-26b are suggested to be a predictive biomarker of major cardiovascular events [66]. Interestingly, Zhang L. et al. found that miR-26b promoted the expression of pro-inflammatory cytokines and chemokines, such as TNF-α, IL-1β, IL-8, and IL-10, and induced NF-κB signalling pathway in bovine alveolar macrophages [67]. Macrophage miR-145 promoted inflammation by activating NF-κB activation, whereas miR-145 downregulation reduced aortic lesion in mice [68]. However, the impact of miR-145 on inflammation is controverted as anti-inflammatory effects of miR-145 have also been reported [69,70]. miR-145 also plays a role in atherosclerosis development, as this microRNA is the most abundant in smooth muscle cells and modulates their plasticity [71]. This miRNA is downregulated in injured carotid arteries, resulting in a lower differentiation of smooth muscle cells [71]. Our study did not allow to elucidate the impact of a beer-mediated decrease in macrophage miR-145 levels on macrophage inflammatory profile or smooth muscle cell differentiation in the atherosclerotic plaque, and further analyses in this regard are guaranteed. Finally, miR-223 was preferentially expressed in myeloid cells, but not in B and T lymphocytes, and regulated myeloid differentiation and macrophage function [72]. miR-223 promoted the polarisation of macrophage towards an M2 anti-inflammatory phenotype and reduced macrophage inflammatory responses activated by TLR signalling [73]. In fact, TLR signalling decreased miR-223 with a consequent increase in the expression of inflammatory cytokines [74]. Therefore, our results showing a decreased expression of miR-223 after non-alcoholic beer consumption could suggest that non-alcoholic beer activates the TLR signalling pathway in macrophages, which is inconsistent with our results showing inhibition of pro-inflammatory microRNAs with non-alcoholic beer. Further studies are needed to clarify the impact of non-alcoholic beer on miR-223 mediated macrophage function. On the other hand, we found an increase in this microRNA after beer consumption. Serum miR-223 levels were higher in alcoholic subjects, compared to controls [75]. In addition, Li et al. found, in a mouse model of chronic ethanol feeding, that miR-223 played a key role in the amelioration of liver injury promoted by alcohol consumption through inhibition of neutrophil infiltration and inflammation in the liver. Thus, the increase in miR-223 that we observed after beer consumption could be due to the alcohol intake [75].

Interestingly, we found that plasma and macrophage miR-30c-5p FCs after beer intake were negatively correlated, whereas plasma and macrophage miR-423-5p FCs after non-alcoholic beer intake were positively correlated. Macrophages deliver microRNAs into exosomes [76]. We cannot assert that the origin of miR-423-5p in circulation is the macrophages, but such a positive correlation suggests that this could be the case.

We found that IX concentration increased after beer intake, both in plasma and urine. IX has been identified as a useful biomarker of beer intake [36]. However, IX levels did not increase significantly after non-alcoholic beer intake. This could be because IX concentration was lower in our samples of non-alcoholic beer. Interestingly, we found a negative correlation between the increase in plasma IX levels and the FC in circulating miR-155-5p and miR-92a-5p levels after non-alcoholic beer intake. It was previously reported that resveratrol, the main red wine polyphenol, decreases the expression of miR-155 in different tissues and experimental models [77,78,79,80]. Other polyphenols like curcumin [81], hydroxytyrosol [82], and oleuropein [83] also decrease miR-155 expression in different cells. miR-92 was also found to be downregulated by green tea polyphenols [84] and diindolylmethane found in cruciferous vegetables [85], and it was postulated that miR-92a mediates the antioncogenic effects of these polyphenols in cancer cell lines. Although more studies are needed to clarify if beer polyphenols are the drivers of microRNA modification and the specific contribution of each polyphenol to the microRNA regulation, our results suggest that miR-155-5p and miR-92a-5p could be modulated by the IX contained in beer.

On the other hand, plasma 8-PG levels did not increase after beer or non-alcoholic beer intake. 8-PG is a prenylated flavonoid with a potent oestrogenic effect that was suggested to have a potential role in the prevention of osteoporosis and cancer in menopausal and postmenopausal women [86]. 8-PG is a secondary metabolite of IX and has a longer half-life than IX, but there are big interindividual differences in the conversion efficiency because this conversion depends on individual factors like the microbiota and the diet [87]. In fact, different conversion phenotypes have been described. In this regard, the study conducted by Bolca et al. in healthy postmenopausal women classified participants into poor, moderate and strong 8-PG producers [88].

Finally, we observed a non-significant decrease in HDL-cholesterol levels after each intervention. This was not in accordance with other studies showing higher HDL-cholesterol levels in wine and beer consumers [89] and a slower decrease in HDL-cholesterol levels in moderate alcohol consumers compared with non-consumers in a longitudinal study [90]. However, the observed decrease in HDL-cholesterol could be due to the significant decrease in MUFA intake during interventions that we observed, as a meta-analysis has shown that higher MUFA intake is related to higher HDL-cholesterol levels [91].

This study has some limitations. First, the small sample size encourages researchers to validate these results in a larger intervention trial including both men and women. Second, although anthropometrical parameters did not change during the intervention, we found some minor dietary changes during intervention phases, such as a lower energy intake due to a lower intake of all macronutrients (carbohydrates, proteins, and fats). However, only the decrease in MUFA intake was significant. This could be due to the so-called “control effect”, which means that participants felt under supervision during the intervention and made unconscious changes in their habitual diet. Given that dietary changes were not significant, we considered that they would not significantly affect our results. However, we must bear in mind that those dietary changes could be a source of bias. Besides, we cannot rule out an impact of beer and non-alcoholic beer consumption on other variables, such as blood pressure, that have not been measured in this study. However, this pilot study set the basis for new, larger intervention trials with a broader population characterisation from a phenotype and a molecular perspective. Finally, to make the intervention protocol more comfortable for participants and, consequently, to increase the adherence to the intervention, we did not restrict the time of day when the beer should be consumed, or the number of beer intakes necessary to consume the defined 500 mL dose. This could lead to more significant variability in microRNA levels. However, this higher variability works against our hypothesis, reducing the likelihood of false positives. Moreover, the intake of both beer and non-alcoholic beer and the compliance with the washout periods were confirmed by measuring IX.

## 5. Conclusions

We showed that the levels of circulating and macrophage microRNAs related to CVD are modulated by beer and non-alcoholic beer consumption with interesting differences between the two types of beer. Our results showed an increase in pro-inflammatory microRNAs with beer and a decrease with non-alcoholic beer. This was in accordance with previous results showing a reduction of the release of inflammatory cytokines by some beer polyphenols and by non-alcoholic beer [92,93,94]. Thus, the different effects of beer and non-alcoholic beer could be due to the different polyphenolic content, as we found different plasma concentrations of IX and 8-PG after consumption of both beers. IX is the major beer polyphenol, but other polyphenols can also be differently concentrated in both types of beer. The differential impact on both beers of some microRNAs could also be due to the alcoholic content. Alcohol consumption can modify the epigenetic mechanism at many levels [95]. In this sense, it was demonstrated that microRNAs are affected by alcohol consumption [96,97].

## Figures and Tables

**Table 1:** Characteristics of participants along the intervention.

	Washout 1	Beer	Washout 2	Non-alcoholic Beer	*p*-Value
Height (m)	1.70 ± 0.05	1.70 ± 0.05	1.70 ± 0.05	1.70 ± 0.05	NS
Weight (kg)	90.36 ± 12.46	90.57 ± 11.84	90.57 ± 11.84	90.69 ± 12.07	NS
BMI (kg/m^2^)	31.071 ± 2.6	31.13 ± 2.61	31.13 ± 2.61	31.03 ± 2.48	NS
Fat mass (%)	32.61 ± 3.33	32.56 ± 4.17	32.56 ± 4.17	32.64 ± 3.5	NS
Lean mass (%)	30.87 ± 1.76	30.63 ± 2.2	30.63 ± 2.2	30.6 ± 1.7	NS
RMR (kcal)	1853.856 ± 177.88	1848.43 ± 169.57	1848.43 ± 169	1850.86 ± 176.17	NS
Visceral fat index	15.86 ± 2.27	15.71 ± 1.98	15.71 ± 1.98	16 ± 2.16	NS
Total cholesterol (mg/dL)	214.614 ± 43.21	203.56 ± 31.28	210.71 ± 34.99	205.94 ± 25.76	NS
HDL-C (mg/dL)	49.19 ± 13.41	45.19 ± 11.71	56.73 ± 21.6	45.64 ± 10.93	NS
LDL-C (mg/dL)	134.63 ± 28.41	123.7 ± 18.93	136.3 ± 24.86	130.38 ± 14.27	NS
Triglycerides (mg/dL)	136.142 ± 108.2	187.43 ± 149.67	132.43 ± 68.88	169.86 ± 165.07	NS
ApoB (mg/dL)	118.3 ± 28.83	112.29 ± 20.52	113.5 ± 21.5	117.83 ± 16.86	NS
CRP (mg/dL)	0.28 ± 0.19	0.23 ± 0.14	0.23 ± 0.13	0.23 ± 0.16	NS
Total energy intake (kcal)	2412.33 ± 450.68	2318.714 ± 419.3	2489.71 ± 406.32	2095.71 ± 383.15	NS
Proteins (g/day)	130.18 ± 34.26	103.56 ± 20.88	118.21 ± 25.65	94.34 ± 28.58	NS
Carbohydrates (g/day)	252.5 ± 49.81	248.43 ± 53.42	266.71 ± 61.89	250.29 ± 43.14	NS
Sugars (g/day)	112.6 ± 27.32	111.97 ± 36.34	121.83 ± 29.41	123.51 ± 19.78	NS
Starch (g/day)	109.67 ± 29.94	122.39 ± 29.19	120.86 ± 18.2	112.1 ± 32.38	NS
Fibre (g/day)	18.683 ± 5.95	20.14 ± 3.96	20.7 ± 7.55	20.51 ± 8.47	NS
Fats (g/day)	91.63 ± 19.5	81.5 ± 27.32	96 ± 23.23	70.7 ± 18.71	NS
SFA (g/day)	34.85 ± 9.98	29.24 ± 14.03	34.47 ± 12	25.59 ± 7.27	NS
MUFA (g/day)	34.15 ± 7.19	26.33 ± 7.2	33.74 ± 10.39	22.01 ± 4.87	0.02
PUFA (g/day)	11.23 ± 0.86	13.242 ± 5.31	14.1 ± 1.93	10.27 ± 4.59	NS

NS = non-significant. RMR = resting metabolic rate. BMI = body mass index. SFA = saturated fatty acids; MUFA = mono-unsaturated fatty acids; PUFA = polyunsaturated fatty acids. Shown is the mean ± SD. *p*-value refers to the comparison between collection timepoints and was calculated with repeated measurements ANOVA.

## Data Availability

There are restrictions on the availability due to the signed consent agreements around data sharing, which only allow access to external researchers for studies following the project purposes. Requestors wishing to access the data used in this study can make a request to the corresponding author.

## Supplementary Materials

The following are available online at https://www.mdpi.com/2072-6643/13/1/69/s1, Figure S1: Recruitment and intervention workflow and design. Figure S2: Levels of isoxanthohumol (IX) and 8-prenylnaringenin (8-PG) in beer and non-alcoholic beer samples. Figure S3: Correlation between plasma miR-320a-3p and LDL-cholesterol levels. Figure S4: Effect of beer and non-alcoholic beer interventions on *DENND5B* and *MDM2* expression. Figure S5: Correlation between FC in circulating miR-155-5p and miR-92a-5p levels and FC in plasma IX and 8-PG levels after non-alcoholic beer intake. Table S1: List of selected miRNAs. Table S2: List of genes targeted by plasma microRNAs modulated by beer intake. Table S3: List of genes targeted by macrophage microRNAs modulated by beer intake.
