# Supplementary figures and images for: Alcoholic and Non-Alcoholic Beer Modulate Plasma and Macrophage microRNAs Differently in a Pilot Intervention in Humans with Cardiovascular Risk
Source: Nutrients. 2020 Dec 28;13(1):69. doi: 10.3390/nu13010069 (PMC7823561; doi:10.3390/nu13010069)

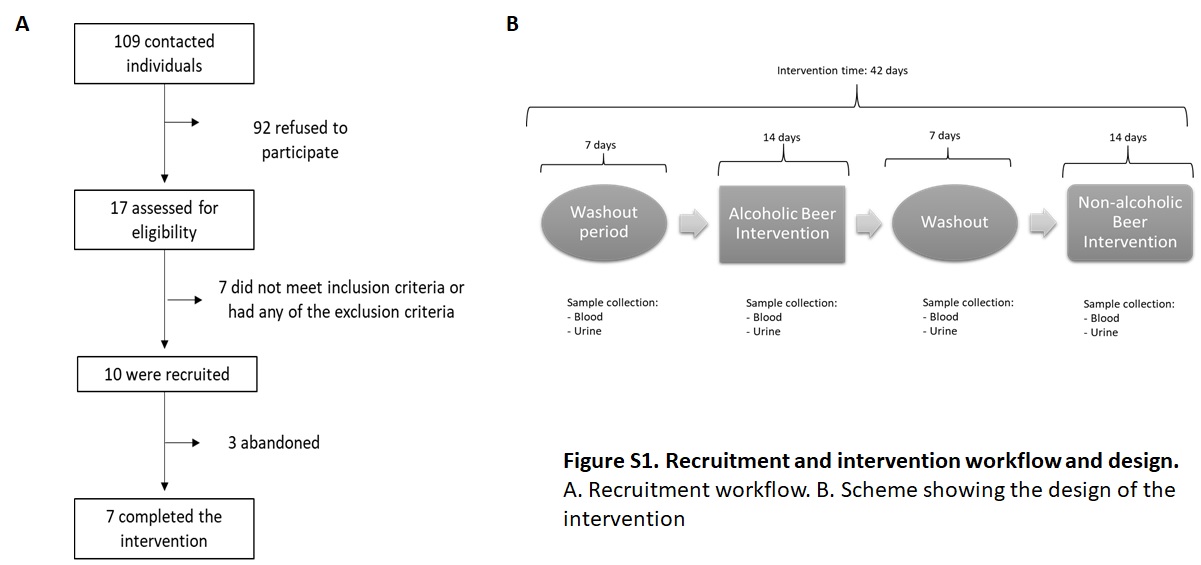

Supplement: Supplementary file 1 [file nutrients-13-00069-s001.zip › Figure S1.jpg]

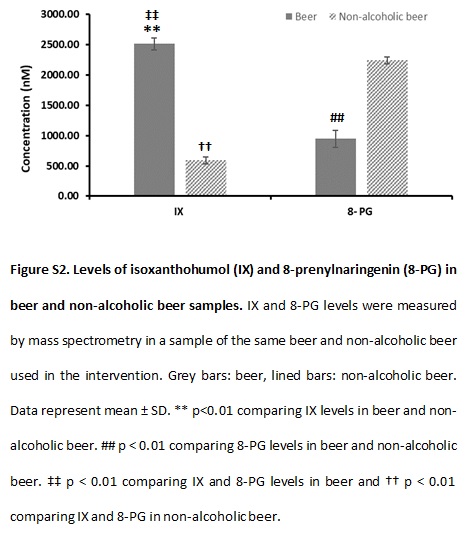

Supplement: Supplementary file 1 [file nutrients-13-00069-s001.zip › Figure S2.jpg]

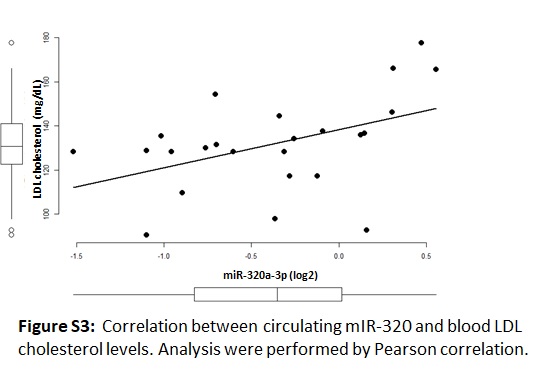

Supplement: Supplementary file 1 [file nutrients-13-00069-s001.zip › Figure S3.jpg]

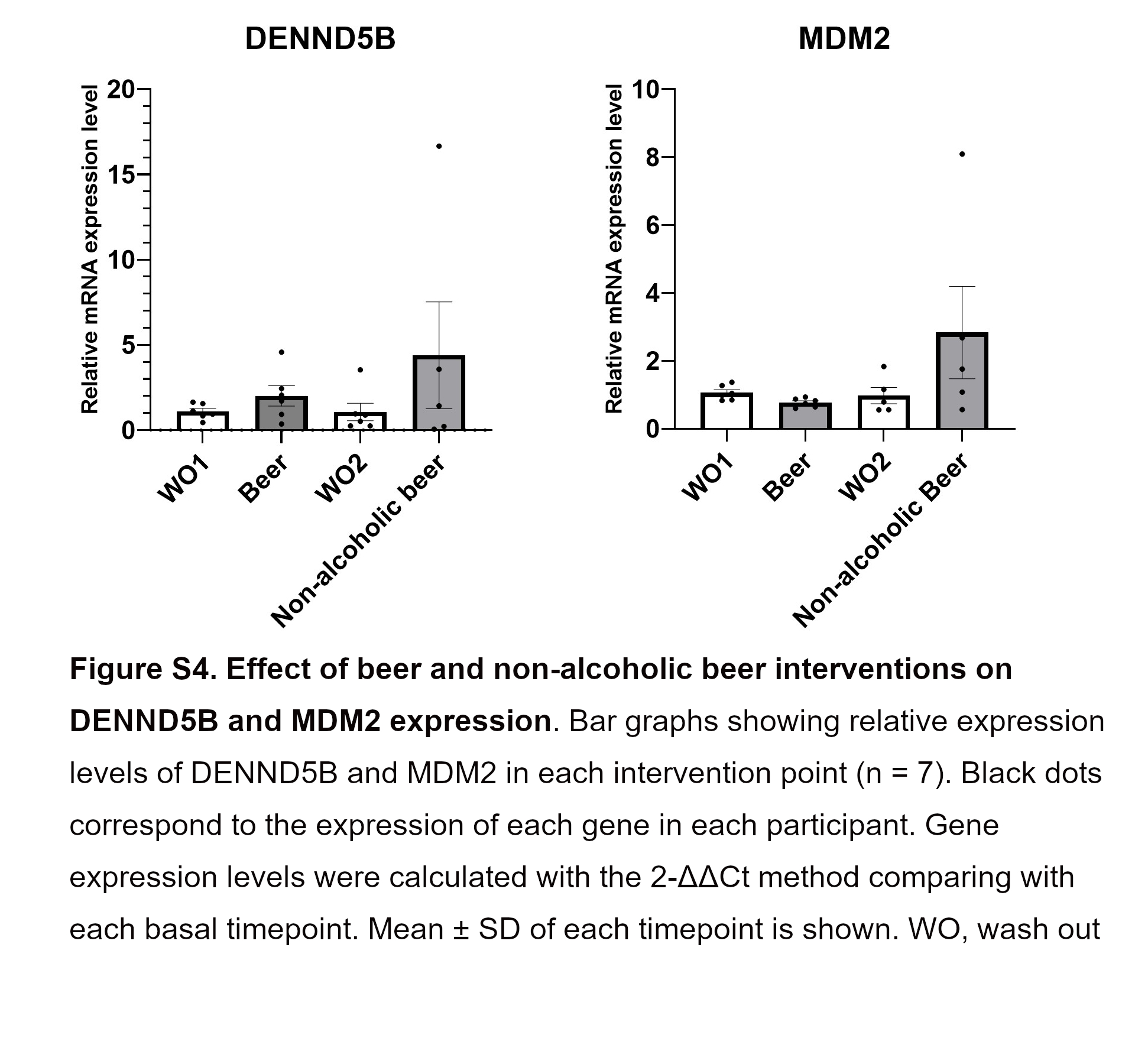

Supplement: Supplementary file 1 [file nutrients-13-00069-s001.zip › Figure S4.jpg]

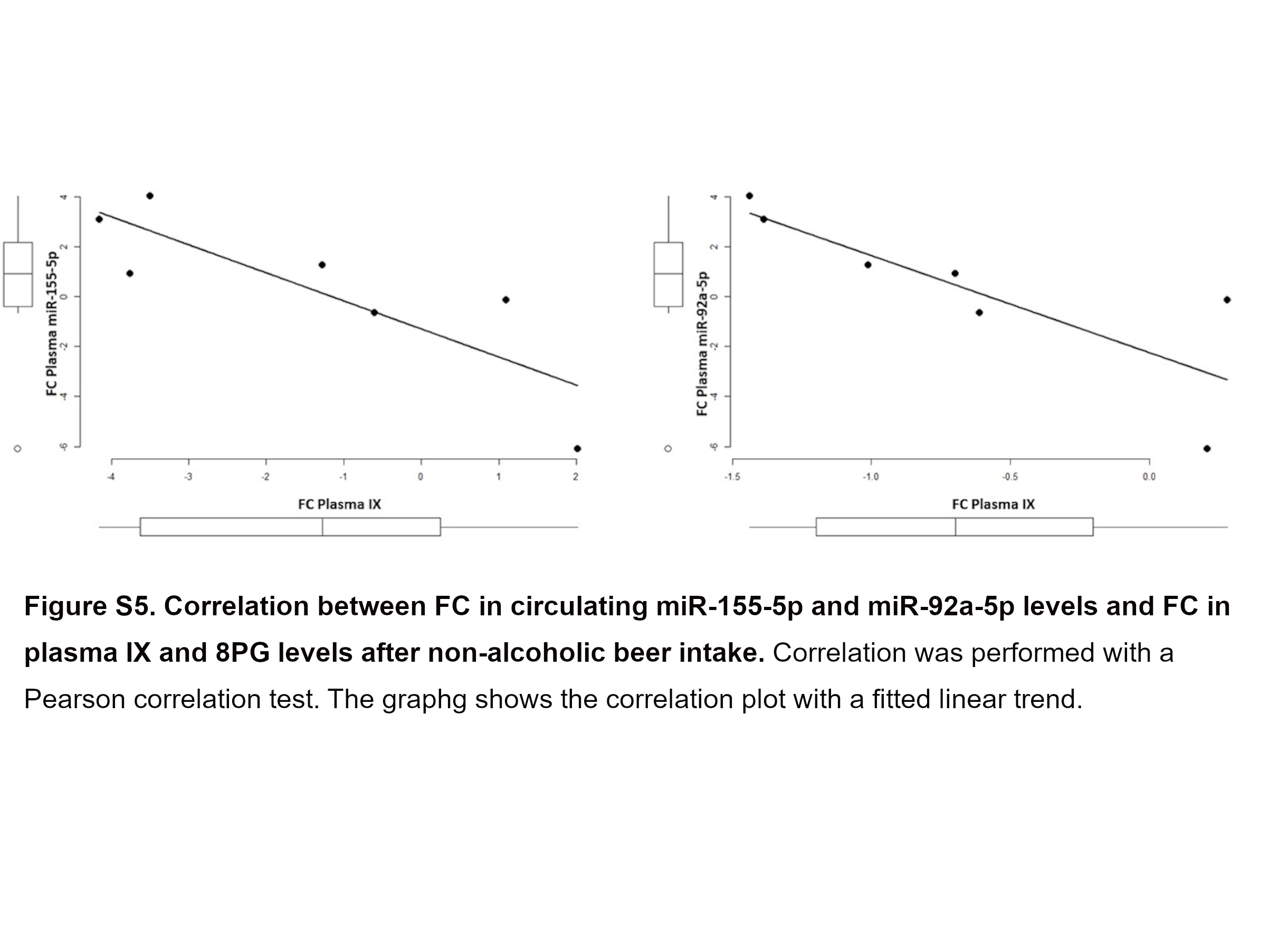

Supplement: Supplementary file 1 [file nutrients-13-00069-s001.zip › Figure S5.jpg]
